# Mindfulness meditation modulates stress-eating and its neural correlates

**DOI:** 10.1038/s41598-024-57687-7

**Published:** 2024-03-27

**Authors:** Alyssa Torske, Benno Bremer, Britta Karen Hölzel, Alexander Maczka, Kathrin Koch

**Affiliations:** 1 Department of Diagnostic and Interventional Neuroradiology, School of Medicine and Health, Technical University of Munich, Munich, Germany; 2 TUM-Neuroimaging Center (TUM-NIC), Klinikum Rechts Der Isar, Technical University of Munich, Munich, Germany; 3 Graduate School of Systemic Neurosciences, Ludwig Maximilians Universität München, Martinsried, Germany

**Keywords:** Mindfulness, Stress, Eating-Behavior, fMRI, Resting state functional connectivity, Human behaviour, Psychology, Neuroscience, Feeding behaviour

## Abstract

Stress-related overeating can lead to excessive weight gain, increasing the risk of metabolic and cardiovascular disease. Mindfulness meditation has been demonstrated to reduce stress and increase interoceptive awareness and could, therefore, be an effective intervention for stress-related overeating behavior. To investigate the effects of mindfulness meditation on stress-eating behavior, meditation-naïve individuals with a tendency to stress-eat (*N* = 66) participated in either a 31-day, web-based mindfulness meditation training or a health training condition. Behavioral and resting-state fMRI data were acquired before and after the intervention. Mindfulness meditation training, in comparison to health training, was found to significantly increase mindfulness while simultaneously reducing stress- and emotional-eating tendencies as well as food cravings. These behavioral results were accompanied by functional connectivity changes between the hypothalamus, reward regions, and several areas of the default mode network in addition to changes observed between the insula and somatosensory areas. Additional changes between seed regions (i.e., hypothalamus and insula) and brain areas attributed to emotion regulation, awareness, attention, and sensory integration were observed. Notably, these changes in functional connectivity correlated with behavioral changes, thereby providing insight into the underlying neural mechanisms of the effects of mindfulness on stress-eating.

Clinical trial on the ISRCTN registry: trial ID ISRCTN12901054

## Introduction

Chronic stress has been demonstrated to have negative implications on many health-related domains, including eating behavior. This is due, in part, to the physiological stress response, which includes the sympathetic and parasympathetic nervous systems in addition to the hypothalamic-pituitary-adrenal (HPA) axis^1,2^. While these systems work together to allow the body to effectively respond to acute stress, chronic stress can lead to the dysregulation of the HPA axis, which can result in the aberrant production of stress hormones^1,3^. This can, in turn, affect the production of leptin, ghrelin, and neuropeptide Y, which play an integral role in regulating perceived hunger and satiety^1,2,4–7^. Chronic stress can, therefore, increase feelings of hunger and increase caloric intake^8–12^.

Furthermore, chronic stress has been shown to implicate executive functioning including emotion regulation and cognitive control^13^. This can have a significant effect on decision-making abilities as well as influence impulsive snacking and emotional eating behaviors^13–15^. This is particularly noteworthy as a large portion of the global population lives in an obesogenic environment, i.e., individuals are persistently being exposed to high-calorie food items^16–18^. Given that the mere presence of high-calorie food and its odors can elicit appetite-enhancing effects ^19^, stressed individuals may not only be more prone to experiencing an increase in appetite through the dysregulation of their HPA axis but may also be less able to utilize cognitive control (i.e., restraint) when encountering high-calorie foods.

Ultimately, the long-term effects of chronic stress and its influence on eating behavior can have negative implications on metabolic and cardiovascular health. For example, frequently engaging in stress-related overeating behavior (and the subsequent weight gain) can lead to type 2 diabetes and hypertensionor other cardiovascular diseases^12,20–22^. Considering the profound effect stress has on eating behavior, its long-term health consequences, in addition to the high global prevalence of cardiovascular and metabolic disease^23–25^, it is crucial for the scientific community to establish and investigate stress-related overeating behavior interventions.

To this end, mindfulness meditation training (MMT) has been demonstrated to be a reliable stress-reduction mechanism that can also improve overall well-being^26–32^. MMT involves the intentional focus of attention as well as open awareness of the present moment without judgment or distraction^33^. Through its ability to bring attention to thoughts, feelings, and bodily sensations, MMT can support individuals in developing greater interoceptive awareness, emotion regulation, and reduce stress sensitivity^27,34–39^. By becoming more aware of thoughts and emotions, individuals can observe stressors with greater objectivity and compassion, thereby reducing the physiological stress response in addition to the susceptibility to chronic stress^40–42^. Given the role MMT plays in reducing perceived stress as well as the physiological stress response, it is hypothesized that individuals suffering from stress-related overeating behavior would benefit from a food-related MMT.

As both stress and mindfulness have an effect on the neural level^29,43–47^, this study seeks to investigate the neural mechanisms through which MMT influences stress-eating behavior via seed-based resting-state functional connectivity (FC) changes. As the hypothalamus and insula are the fundamental regions in the mediation of the neural processes of hunger and satiety cues, these brain areas were selected as a-priori seed regions of interest.

The hypothalamus and its individual nuclei, through its involvement in the HPA axis, initiates change in appetite and food intake^2,4–7^. While the lateral hypothalamus, through the release of hormones such as neuropeptide Y and agouti-related peptides, regulates perceived hunger^46,48–50^, the medial hypothalamus regulates perceived satiety through the release of hormones such as melanin-concentrating-hormone (MCH)^50^.

Furthermore, a review published by Syan, McIntyre-Wood^46^ could demonstrate an increase in FC between the medial hypothalamus and areas of the reward system, suggesting an increased interdependence between perceived reward and satiety in individuals with obesity while additionally demonstrating that, in comparison to controls, individuals with obesity exhibited an increase in FC between the lateral hypothalamus and somatosensory areas. These findings suggest that differences in eating behavior are accompanied by specific FC changes in the brain. Therefore, given its central role in the complex interplay between stress, appetite, and eating behavior, the hypothalamus was selected as a region to observe possible neuronal changes elicited by food-related MMT.

The insula is a brain area that contributes to cognitive processes underlying both MMT and eating behavior. For example, the insula has been demonstrated to be involved in interoception and attentional control, which are both fundamental aspects of MMT as well as eating behavior^51^. Moreover, the insula and its nuclei play a role in the subjective experience of flavor, texture, and smell^52–54^, which contribute to the hedonic evaluation of food. For example, MMT and its involvement in fostering awareness has been associated with the anterior insula, an area involved in emotion regulation and interoceptive awareness^55–57^, which are two factors relevant to eating behavior, hunger, and satiety cues, thereby allowing for or less emotion-dependent food selections^51,58^. The posterior insula, on the other hand, is associated with sensory processing and the integration of taste-related stimuli, which ultimately influence food choice^59^. The insula is, therefore, not only associated with regulating perceived hunger and satiety cues but also plays a fundamental role in mediating cognitive, emotional, and sensory processes related to both mindfulness and stress-related eating behavior.

This research investigating the FC changes between the hypothalamus, insula, and the whole brain will not only provide insight into the effects of MMT on stress-related eating behavior but also into the neural mechanisms underlying eating behavior and stress reduction. These results could, therefore, provide critical evidence of an intervention for stress-eaters to cultivate a more mindful relationship with food.

## Methods

### Participants

Addressing individuals with a tendency to overeat when stressed, participants were recruited via the university hospital’s mailing list and online advertisements. Participants were required to report moderate to high levels of stress, as assessed by the Perceived Stress Scale (PSS)^60^ while expressing a tendency to engage in stress-eating and needed to fulfill the following criteria: (1) between the ages of 18 and 45 (2) general MRI suitability (i.e., no metal implants and not prone to claustrophobia), (3) body-mass-index (BMI) between 18 and 30, (4) no dietary restrictions (including vegetarianism or veganism)^61^, (5) no use of oral contraceptives or intrauterine devices, (6) no known, untreated, thyroid dysfunction, (7) no chronic respiratory diseases. All participants provided written, informed, consent and were given monetary compensation for their participation. This study is listed as a clinical trial on the ISRCTN registry with trial ID ISRCTN12901054 registered on 19/05/2023, was performed in accordance with the Declaration of Helsinki, and was approved by the Ethics Committee of Klinikum Rechts der Isar, Technical University Munich.

### Procedure

This study was designed as a pseudo-randomized, actively controled trial to investigate the effects of MMT on stress-eating behavior and its neural correlates. Participants were single-blindedly (subject only) allocated to either the MMT condition or the active control, health training (HT) condition. All participants underwent magnetic resonance imaging (MRI), and psychometric testing evaluating perceived mindfulness, stress, emotional eating, food cravings, dietary restraint, and the assessment of body weight prior to and after completing the intervention. All measures were acquired after a subjectively stressful day and participants were instructed to abstain from eating five hours prior to their scheduled measure. The training programs for both conditions were accessible via an online platform and consisted of 31 daily 15 min sessions. To promote training adherence, daily reminders were sent to participants by email.

The MMT was developed in close cooperation with author BKH who is a certified MBSR instructor. In the MMT, participants were provided with a detailed introduction to the theoretical framework of MMT while additionally guiding participants through the daily meditation exercises via video or audio clips. Written instructions emphasized the relationship between MMT and eating behavior, encouraging participants to engage more mindfully with food, for example, by consciously attending to the flavor and texture of the food they consume or to any emotions that arise in the context of eating. The HT condition provided participants with informative, health-related video and audio clip excerpts from popular science broadcasting networks in Germany. Designed to imitate the MMT, the HT was delivered via the same online platform and followed the same order of video and audio material. However, topics in the HT did not include any information pertaining to mindfulness, eating behavior, or nutrition. For a detailed description of the training content, please see Table S1. Participants were required to complete at least 27 training sessions to be included in the final analysis.

**Figure 1:**
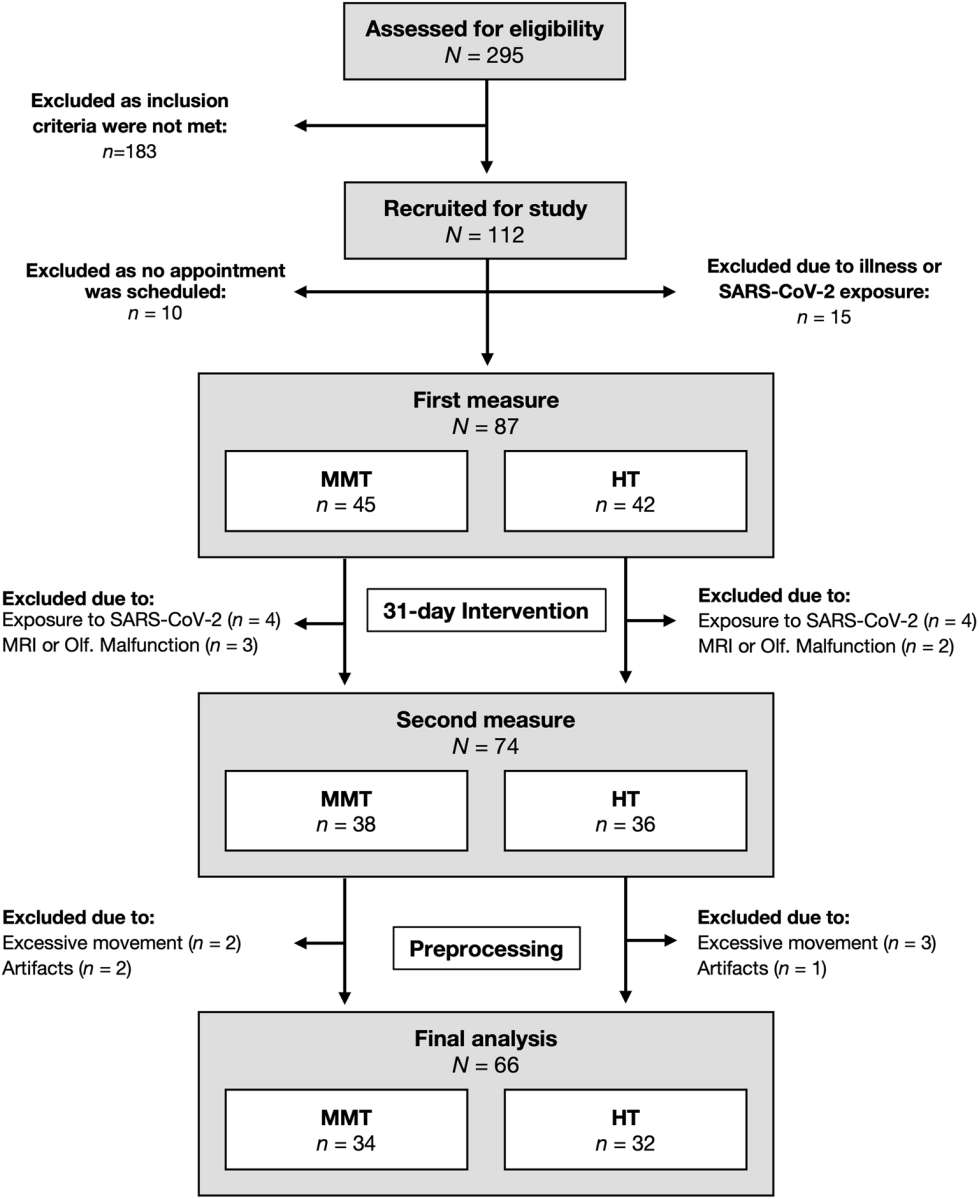
Data acquisition process (June 2019—June 2021). *MMT* Mindfulness Meditation Training, *HT* Health Training.

295 participants were assessed for eligibility, and 112 participants fulfilled the inclusion criteria. 87 participants completed the first MRI measure, and 74 participants were also available for the second MRI measure. After preprocessing, data from 66 participants (33 female) were included in the final analysis. Data acquisition took place between June 2019 and June 2021 and is depicted in Fig. 1.

To verify the pseudo-randomization process, final samples were compared for demographic characteristics, measuring intervals, and an average number of sessions completed using *t*-tests for independent samples or chi-square tests, respectively.

### Behavioral data

To investigate various behavioral measures pertaining to the effects of MMT on eating behavior, participants completed the German version of the Mindful Attention and Awareness Scale (MAAS)^62^, Food Cravings Questionnaire-Trait (FCQ-T), Salzburg Stress Eating Scale (SSES), Salzburg Emotional Eating Scale (SEES), as well the Restraint scale.

The statistical analyses of behavioral data were performed using SPSS v29.0. Independent *t*-tests were conducted on data obtained during the first measure to identify and exclude potential baseline differences. To assess training effects, psychometric data was entered into a 2 × 2 mixed effects analysis of variance (ANOVA), where group was the between-subject factor and time was the within-subject factor. Results were thresholded at *p* < 0.05.

### MRI data acquisition

MRI data were acquired on a 3 T Philips MRI scanner with a 32-channel head coil at Klinikum Rechts der Isar’s department of neuroradiology in Munich, Germany.

T2*-weighted resting-state functional images were acquired using echo planar imaging (EPI) with the following scanning parameters: Multiband (MB) factor 2, repetition time (TR) 2.3 s, echo time (TE) 30 ms and flip angle 75°. The field of view (FOV) was set to (192 × 192 × 136) mm^3^, corresponding to a matrix size of 96 × 96 with 62 axial slices of 2 × 2 × 2 mm^3^ large isotropic voxels. 250 volumes were obtained over the course of approximately 10 min. Subjects were instructed to keep their eyes closed, to refrain from engaging in any trains of thought as much as possible, and to not fall asleep.

Additional high-resolution T1-weighted anatomical images were acquired using an MPRAGE sequence with the following scanning parameters: TR 11 ms, TE 5.2 ms and flip angle 8°. 230 axial AC-PC slices encompassing a 384 × 384 matrix of 0.7 × 0.7 × 0.7 mm3 large isotropic voxels were obtained. All anatomical images underwent clinical inspection by a neuroradiological specialist to detect possible structural pathologies.

### Preprocessing

Preprocessing was conducted using SPM 12 (The Wellcome Centre for Human Neuroimaging; http://www.fil.ion.ucl.ac.uk/spm). The preprocessing pipeline was created using RestPLUS^63^ during which the anatomical image was coregistered to the mean functional image and segmented into tissue probability maps used to create a group-specific DARTEL template^64^. Using these templates, the realigned functional time-series were normalized to MNI space and smoothed using a 4 × 4 × 4 mm^3^ full width at half maximum (FWHM) Gaussian Kernel. Additional preprocessing steps were performed using the CONN Toolbox v21.a^65^ and included denoising by regressing out white matter and cerebrospinal fluid using CompCor^66^, filtering time courses with a bandpass filter of 0.01 to 0.1 Hz, as well as de-trending and de-spiking.

### Seed-based functional connectivity

Prior evidence demonstrated the differential involvement of hypothalamic and insular subnuclei. Therefore, bihemispheric medial and lateral hypothalamus or the anterior and posterior insula seeds were utilized. Canonical parcellations of the hypothalamus were extracted from the WFU PickAtlas ^67^, while parcellations of the insula were extracted from the Hammers Atlas^68^. Atlas selection was determined by both the availability and anatomic quality of seeds corresponding to our nuclei of interest. It is important to note that since no single atlas provided seed masks for all regions of interest (ROI), seeds from different atlas sources were selected according to their level of establishment and anatomic correspondence.

The seed-based FC analysis was conducted using the CONN Toolbox. First-level connectivity maps using Pearson correlation coefficients between the average time course of voxels within each ROI and every voxel (whole brain) were computed and transformed to z-scores. For the second-level analysis, the resulting FC maps were entered into a 2 × 2 mixed ANOVA, where training group was the between-subject factor and time was the within-subject factor. Results were simultaneously contrasted at MMT > HT and Post > Pre and thresholded at *p* < 0.05, FDR-corrected for multiple comparisons. To evaluate the directionality of the results, both positive and negative contrasts were applied and the *p-* value was adjusted to *p* < (0.05/2 =) 0.025, according to the Bonferroni method.

Correlations between changes in FC and behavioral measures for both MMT and HT groups were assessed. For every subject and time point, a mean signal from each significant cluster was extracted from the first-level maps using DPABI v6.0^69^. The cluster-specific mean signal from the first time point was subtracted from the mean signal of the second time point, resulting in one value per subject which represented the change in cluster-specific FC after the intervention. Changes in behavioral measures were computed by calculating the difference scores (post–pre-intervention). Both values were then entered into a linear regression in SPSS. To avoid overcorrection, we incorporated the number of anatomical regions for multiple comparison corrections, resulting in an adjusted *p*-value of < (0.05/2 =) 0.025.

## Results

### Sample characteristics

Groups did not significantly differ in age, gender, years of education, or BMI (please see Table 1). Participants received their second MRI scan no later than 5 days upon completing the training condition.

**Table 1:** Participant demographics.

	Total (*N* = 66)	MMT (*n* = 34)	HT (*n* = 32)	*p* value
Age, M ± SD	28.0 ± 5.1	27.4 ± 4.9	28.7 ± 5.3	0.30
Female, *n* (%)	33 (50)	17 (50)	16 (50)	1.00
Years of education, M ± SD	18.3 ± 3.0	18.2 ± 3.1	18.4 ± 3.0	0.80
BMI, [kg/m^2^], M ± SD	24.1 ± 4.1	23.4 ± 4.3	25.0 ± 3.9	0.12

### Behavioral data

Groups did not differ significantly in behavioral measures obtained at baseline (see Table S3). Upon completing the training conditions, both groups displayed a slight decline in body weight (MMT: *M*_*Pre*_ = 69.8 kg, *SD* = 2.0; *M*_*Post*_ = 69.4 kg, *SD* = 1.9; HT: *M*_*Pre*_ = 75.8 kg, *SD* = 2.5; *M*_*Post*_ = 75.5 kg, *SD* = 2.6). This change, however, was not statistically significant for either group.

In line with our hypotheses, the MMT was found to effectively increase levels of perceived mindfulness demonstrated via an increase in MAAS (*M*_*Pre*_ = 53.9, *SD* = 10.6; *M*_*Post*_ = 57.9, *SD* = 8.7). No increase in perceived mindfulness was observed in participants of the HT (*M*_*Pre*_ = 55.5, *SD* = 12.6; *M*_*Post*_ = 54.9, *SD* = 11.6). An ANOVA was conducted and determined a significant group-by-time interaction (*F *(1, 64) = 7.74, *p* = 0.007, partial *η*^2^ = 0.108), including a significant main effect of time (*F *(1, 64) = 4.41, *p* = 0.040, partial *η*^2^ = 0.065).

Following the MMT, participants also reported lower FCQ-T scores indicating an overall reduction of food cravings (*M*_*Pre*_ = 84.0, *SD* = 30.3; *M*_*Post*_ = 55.6, *SD* = 27.9). In the HT condition, FCQ-T scores demonstrated no significant difference upon completing the training (*M*_*Pre*_ = 79.4, *SD* = 34.6; *M*_*Post*_ = 78.0, *SD* = 36.0). An ANOVA was conducted and resulted in a significant group-by-time interaction (*F *(1, 64) = 20.60, *p* < 0.001, partial *η*^2^ = 0.243) and a significant main effect of time (*F*(1, 64) = 24.90, *p* < 0.001, partial *η*^2^ = 0.280).

Furthermore, the MMT resulted in a significant reduction of stress- and emotional-eating as assessed by the SSES (*M*_*Pre*_ = 33.7, *SD* = 8.1; *M*_*Post*_ = 31.8, *SD* = 6.7) and SEES (*M*_*Pre*_ = 64.3, *SD* = 7.1; *M*_*Post*_ = 58.4, *SD* = 9.2). Participants of the HT, however, did not demonstrate any significant changes in stress-eating (*M*_*Pre*_ = 32.4, *SD* = 9.9; *M*_*Post*_ = 31.8, *SD* = 10.5) or emotional-eating behavior (*M*_*Pre*_ = 61.8, *SD* = 10.0; *M*_*Post*_ = 60.2, *SD* = 10.7). An ANOVA was conducted and determined a significant group-by-time interaction for both the SSES (*F* (1, 64) = 4.06, *p* = 0.048, partial *η*^2^ = 0.06) and SEES (*F *(1, 64) = 4.94, *p* = 0.030, partial *η*^2^ = 0.072) questionnaires as well as a significant main effect of time for both scales (SSES: *F* (1, 64) = 7.92, *p* = 0.006, partial *η*^2^ = 0.110; SEES: *F* (1, 64) = 15.28, *p* < 0.001, partial *η*^2^ = 0.193). It is important to note that a Levene’s test revealed that the homogeneity of error variances was not given for data pertaining to SSES_Post_ and SEES_Pre_. We therefore repeated the analysis by comparing individual difference scores with *t*-tests for independent samples between groups. Results for the SSES (*t* (64) = 2.02, two-sided* p* = 0.048) and the SEES (*t* (64) = 2.22, two-sided *p* = 0.030) remained consistent, thereby confirming the results of the prior analysis.

No significant group effects (*F* (1, 64) = 3.17, *p* = 0.080, partial *η*^2^ = 0.047) or main effects of time (*F *(1, 64) = 1.26, *p* = 0.267, partial *η*^2^ = 0.019) of the MMT on dietary restraint as measured by the Restraint scale were observed.

**Figure 2:**
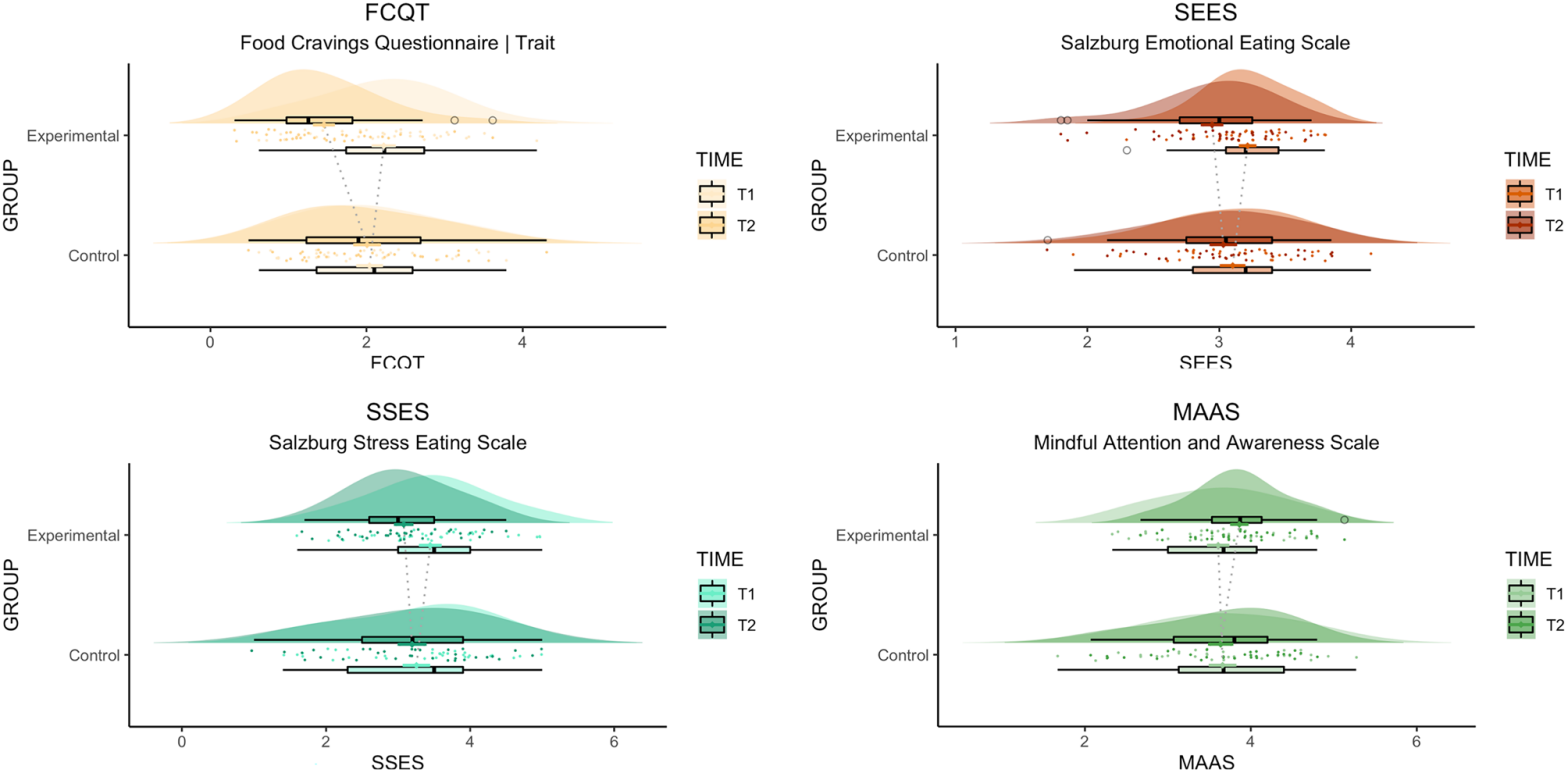
Visualization of the change in various self-report measures between T1 (pre-intervention) and T2 (post-intervention). Experimental group = MMT; Control group = HT.

An overview of the behavioral results can be viewed in Fig. 2.

### Seed-based functional connectivity

The interaction results of the 2 × 2 ANOVA from the whole brain, FDR-corrected analyses revealed significant changes in hypothalamic and insular FC in participants of the MMT group whereas no changes in FC were observed in the HT group, although not all of these changes survived multiple comparisons correction.

The analyses using the left medial hypothalamus as a seed region demonstrated FC increases with two clusters within the right precuneus and an additional cluster within the right angular gyrus. FC analyses using the left medial hypothalamus demonstrated decreases in FC with a cluster extending across the left dorsal striatum and thalamus. The analyses conducted with the right lateral hypothalamus exhibited increased FC with a cluster within the left vPCC, whereas the analyses conducted with the left lateral hypothalamus yielded decreased FC with the left pre-supplementary motor area.

Additional FC increases were observed between the left anterior insula and bilateral clusters within the postcentral gyrus as well as a cluster within the right occipital gyrus. Increased FC was also observed between the left posterior insula and the left postcentral gyrus in addition to observing increases in FC between the right posterior insula and the right inferior parietal lobule.

**Figure 3:**
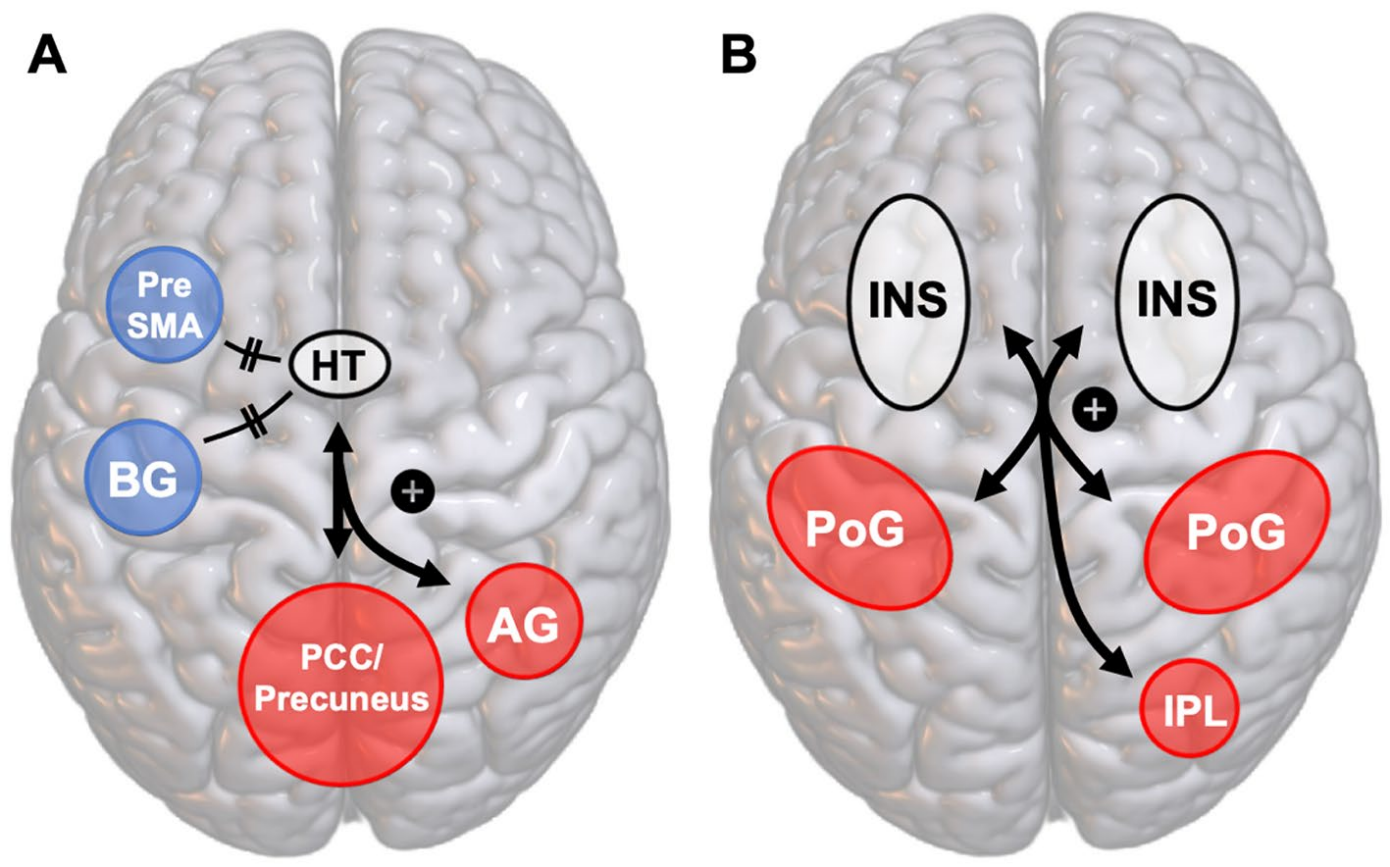
Overview of the FC changes observed between the hypothalamus (**A**), the insula (**B**), and the whole brain. *HT* Hypothalamus, *BG* Basal ganglia, *PreSMA* Pre supplementary motor area. *PCC* Precuneus, *AG* Angular gyrus, *INS* Insula, *PoG* Postcentral gyrus, *IPL* Inferior parietal lobe.

An overview of the FC results can be seen in Table 2 and Fig. 3 and S2.

**Table 2:** ANOVA results with associated anatomic regions.

ROI		FC	Cluster region	Peak MNI coordinates			*k*	*p*_FWE_	*p*_FDR_
				*x*	*y*	*z*			
Hypothalamus	L. Lateral	↓	L. PreSMA	− 18	10	60	49	0.047	0.042
	R. Lateral	↑	L. Ventral PCC	− 14	− 58	10	131	0.081	0.027
	L. Medial	↓	L. Striatum/Thalamus	− 16	− 16	− 4	137	0.03	0.033
	L. Medial	↑	R. Precuneus	4	− 82	34	297	0.00025	0.00023
	L. Medial	↑	R. Caudal Precuneus	8	− 52	64	119	0.15	0.033
	L. Medial	↑	R. Angular Gyrus	46	− 46	16	128	0.10	0.037
Insula	L. Anterior	↑	L. Postcentral Gyrus	− 52	− 20	46	329	0.00002	0.000001
	L. Anterior	↑	R. Postcentral gyrus	48	− 74	− 6	150	0.07	0.03
	L. Anterior	↑	Temporal Lobe / Occipital Cortex	44	− 26	− 52	143	0.10	0.02
	L. Posterior	↑	L. Postcentral Gyrus	− 46	− 30	48	167	0.046	0.034
	R. Posterior	↑	R. Inferior Parietal Lobe	48	− 32	32	337	0.003	0.002

Arrows depict directionality of the changes of functional connectivity (↑ increase and ↓ decrease).

*FC* functional connectivity, *k* Number of voxels in cluster,* R* Right hemispheric, *L* Left hemispheric, *PreSMA* Pre-supplementary motor area, *PCC* Posterior cingulate cortex.

**Figure 4:**
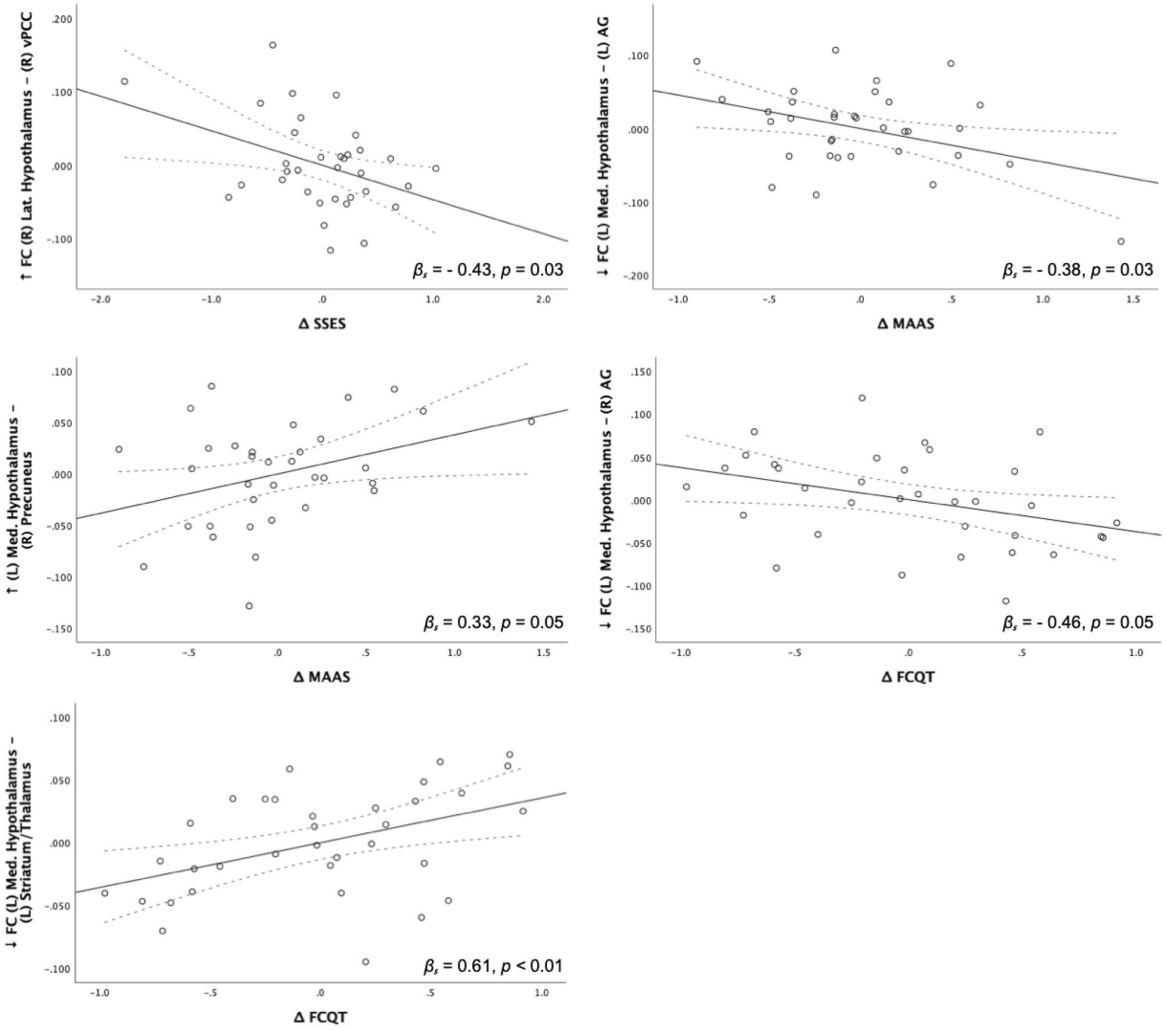
Scatter plots of significant and trending results of the linear regression between the change in functional connectivity and the change in behavioral measures. Dashed lines represent the 95% confidence intervals. *FC* Functional connectivity, *R* Right hemispheric, *L* Left hemispheric, *lat*.  lateral, *med*. medial, *AG* Angular gyrus, *vPCC* Ventral posterior cingulate cortex, *FCQT* Food Cravings Questionnaire—trait, *MAAS* Mindful Attention and Awareness Scale, *SSES* Salzburg Stress Eating Scale).

Interestingly, changes in hypothalamic connectivity patterns in the MMT group (but not the HT group) were accompanied by a multitude of changes on the behavioral level (see Fig. 4 and Table S4). For example, greater decoupling of FC between the left medial hypothalamus and the left dorsal striatum and thalamus significantly correlated with a greater reduction of FCQ-T scores. Additionally, SSES scores were inversely correlated with an increased FC between the right lateral hypothalamus and the left vPCC. These results indicate that the greater the decline in stress eating behavior, the greater the increase in FC between the lateral hypothalamus and the vPCC was observed. Furthermore, our results demonstrated that increases in MAAS score exhibited a trending towards significant with increased FC between the left medial hypothalamus and the right precuneus, whereas a negative correlation between MAAS score and an increase in FC between the left medial hypothalamus and the right angular gyrus was observed.

It is important to note that FC changes of the insula did not correlate with any of the changes of behavioral measures, nor were any significant correlations observed between FC changes and behavioral measures of the HT group.

## Discussion

This study could successfully demonstrate that MMT reduces stress-eating tendencies while also increasing perceived mindfulness. In fact, the observed behavioral changes significantly correlated with FC alterations demonstrating increased and decreased coupling of brain areas relevant to eating behavior, self-referential thinking, mind-wandering, reward perception, and the processing of sensory stimuli.

### Self-Report Measures

Our results demonstrate a significant reduction in perceived stress-eating, emotional eating, and food cravings as well as an increase in perceived mindfulness in daily life. Our findings are in line with prior observations supporting the positive effects MMT has on stress, emotion regulation, interoceptive awareness, and perceived mindfulness^70–75^.

### Functional connectivity measures

#### Hypothalamus

The results of this study demonstrate an increase in FC between subregions of the hypothalamus and multiple clusters within the precuneus, vPCC, and angular gyrus in the MMT group. Interestingly, these clusters pertain to hub regions of the DMN. The DMN is associated with self-referential thinking and mind-wandering^76^ and has been implicated in prior mindfulness research. For example, in comparison to meditation-naïve individuals, studies could demonstrate that experienced meditators yielded less connectivity between hubs of the DMN, which indicates an overall reduction of network activation^44^. In addition, a growing body of evidence suggests that MMT increases connectivity between the DMN and other networks, especially the salience network (SN)^43,77^, whose hub regions process emotional and sensory information^76^. These connectivity increases could be interpreted as an increase in awareness as a result of MMT^43^. Similarly, increased connectivity between hub regions of the DMN and the hypothalamus could indicate that MMT facilitates the perception of hunger and satiety, which could reflect increased interoceptive awareness, a cognitive process fundamental in the regulation of eating behavior.

Remarkably, these findings were corroborated by correlations observed between alterations in FC and behavioral changes. For example, increased FC between the medial hypothalamus and the precuneus positively correlated with an increase in MAAS scores. Therefore, when individuals perceive themselves to be more mindful, satiety cues may be more strongly linked with self-referential processing. An additional negative correlation between an increase in FC between the lateral hypothalamus and the vPCC with the reduction in SSES scores was observed. This suggests that the greater the increase in FC between these brain areas, the less participants perceived their tendency to succumb to stress-eating. Taken together, our findings indicate that MMT strengthens the interaction between hub regions of the DMN and the hypothalamus which goes along with increased levels of perceived mindfulness and a reduction in self-reported stress-eating behavior.

We could also observe MMT’s influence on the reward system through a reduction in FC between the left medial hypothalamus and the left dorsal striatum. The dorsal striatum has been demonstrated to elicit cravings and reward-seeking behaviors^78^. Therefore, a reduction in FC between the medial hypothalamus and the dorsal striatum could indicate a decoupling of brain areas responsible for processing feelings of satiety and reward. Notably, a significant, positive, correlation between the observed reduction in FC between these two brain areas and the decrease in FCQ-T scores was observed. This indicates that a greater reduction in FC between the lateral hypothalamus and the dorsal striatum was associated with a greater reduction in food cravings in participants of the MMT group. These results imply that feelings of satiety may be perceived as less rewarding, which could consequently reduce the tendency to develop food cravings.

Moreover, we could observe FC increases between the medial hypothalamus and the angular gyrus (AG) which is said to play a role in the integration of sensory information relevant to the processing of the smell and taste of food^79^. Interestingly, these connectivity increments negatively correlated with the reduction in perceived food cravings (via the FCQ-T) following the MMT. This indicates that MMT strengthens connectivity between areas involved in the processing of perceived satiety and integrating food-related sensory perception which goes along with a reduced tendency of displaying food cravings.

In addition, our results demonstrate alterations in FC between the left lateral hypothalamus and the left PreSMA, a brain region known to regulate automatic movement and reward behavior^80,81^. Our results may indicate that MMT reduces automatic, action-oriented behaviors when hunger is perceived. This could be attributed to more reflective decision-making about perceived hunger elicited via MMT prior to engaging in goal-directed behavior to find or eat food.

Overall, the findings pertaining to the hypothalamus support MMT’s role in cultivating a more mindful relationship with food. A recent systematic review conducted by Syan, McIntyre-Wood^46^ aimed to characterize hypothalamic resting state FC patterns in individuals with obesity in comparison to controls. Authors could identify patterns of aberrant DMN connectivity (i.e., hypoconnectivity), as well as increases in FC between the hypothalamus and regions attributed with limbic regions associated with reward processing. Interestingly, the MMT utilized in the present study induced opposing changes, i.e., increased hypothalamic connectivity with hubs of the DMN and a decrease of FC with limbic regions. This suggests that MMT might be able to counteract obesity-related neuronal activation patterns. In addition to our results demonstrating sensory integration and automatic eating behavior, these findings indicate that MMT might be able to beneficially influence neural correlates of stress-related eating behavior.

#### Insula

Our results demonstrated an increase in FC between both the anterior and the posterior division of the insula and bilateral clusters within the postcentral gyrus in the MMT-group. The postcentral gyrus, or primary somatosensory cortex, receives sensory information from the periphery; sensory input is organized topographically with tactile information originating from facial and oral sensations processed in the lateral postcentral gyrus which is where the clusters were observed^82^.

Given the emphasis MMT puts on the conscious perception of bodily, especially oral sensations, it seems plausible that areas responsible for perceiving somatosensory sensations in addition to areas that integrate the sensations into awareness are simultaneously activated. As it is known that regions and networks involved in sensory processing are stably active (also during rest), participants of the food-related MMT could demonstrate an increase in activation and connectivity within and between sensory areas pertaining to eating behavior at rest. In addition, the insula has been identified as part of the primary gustatory cortex, therefore responsible for the perception of taste^83^. An increase in the interaction between areas of the primary gustatory cortex and the postcentral gyrus suggest an increased integration of the sensory components, i.e., texture and taste, involved in the sensation of eating. Additional connectivity increases between the posterior insula and the supramarginal gyrus, an area associated with the somatosensory cortex, provide further evidence for the MMT-elicited integration of components involved in the processing of sensations. These results indicate that a food-related MMT can increase the efficiency of sensory integration all while facilitating increased awareness.

Given that the FC changes observed together with the insula involved brain regions essential in sensory processing and integration, a plausible explanation as to why no significant correlations between FC and self-report measures were observed may be because our self-report measures did not account for changes in sensory perception. Nevertheless, these results provide insight into MMT’s ability to alter FC pertaining specifically to the processing of sensory stimuli, which can consequently alter eating behavior.

Overall, the MMT-associated FC changes provide evidence of MMT’s ability to alter resting-state FC pertaining to regions of the DMN, reward perception, and the processing of sensory stimuli, in addition to providing evidence of MMT’s ability to reduce stress- and emotional-eating tendencies. Corresponding to our initial hypotheses, these results suggest that MMT might be an effective intervention strategy for potentially harmful eating behaviors, but more research is needed to confirm this. Moreover, following high methodological standards, this study was able to demonstrate that these changes are accompanied by specific alterations in brain function, thereby contributing to our understanding of the beneficial effects of mindfulness meditation.

#### Limitations

The results of this study should be interpreted under the consideration of methodological limitations. First, given our hypothesis-driven, a-priori selection of seed region, approach, our results may overlook relevant interactions. Nevertheless, the existing literature suggests that the hypothalamus and insula provide a relevant perspective on both mindfulness and eating behavior. Therefore, we believe that the results generated from the present study allow for a better understanding of the mechanisms underlying the intersection of mindfulness and eating behavior. While we acknowledge the complex nature of both eating behavior and MMT, and therefore do not rule out the possibility of further relevant influences, data-driven methods, e.g., independent component analyses should be utilized to further investigate its effects.

Future studies should also consider the influence of other potentially relevant variables, such as BMI, on susceptibility to MMT or similar interventions, e.g., by stratifying the sample by these variables. This could yield useful information for adjusting these interventions to individual requirements and, thereby, potentially improve their effectiveness.

Moreover, it is important to note that not all the results withstood multiple comparison corrections. While it would be a considerable alternative to make the thresholding more liberal, the recurrent patterns across ROIs, along with the statistical interdependence with behavioral measures, give sufficient grounds to assume the plausibility of these results.

Finally, stress-eating was measured using self-report, i.e., indirect, behavioral measures. While we acknowledge that these measures are not an immediate representation of behavior, we believe that the fact that we observed consistent changes across various scales demonstrates the ability of MMT to beneficially influence stress-related overeating behavior. A more immediate estimate of behavior could be better observed through fMRI with task-based paradigms. However, the functional organization of the brain during rest provides the foundation for behavior, i.e., through the formation of continuously activated or inactivated neuronal interaction patterns. Resting-state fMRI, therefore, not only makes it possible to investigate the neural foundation of stress-eating behavior but also allows for a broader perspective beyond a task-dependent state. Hence, we believe, that, despite their individual limitation, the synthesis of our behavioral and resting-state fMRI results conveys valuable information on how MMT influences the functional architecture of the brain and, thereby, cultivates a healthier relationship with food.

### Supplementary Information

Supplementary Information.

## Data Availability

Data have been made publicly available via the Open Science Framework at https://osf.io/pf3gv/.
