# Supplementary material for: Mindfulness meditation modulates stress-eating and its neural correlates
Source: Sci Rep. 2024 Mar 27;14:7294. doi: 10.1038/s41598-024-57687-7 (PMC10973375; doi:10.1038/s41598-024-57687-7)
Supplement: Supplementary file 1 — Supplementary Information. [file 41598_2024_57687_MOESM1_ESM.docx]

**Supplementary material**

|  |  | **MMT** |  | **HT** |
| --- | --- | --- | --- | --- |
| **Session** | **Format** | **Theoretical training** | **Practical training** | **Theoretical training** |
| **1** | Video | Introduction to mindfulness | Mindful breathing A | Sleep |
| **2** | Audio | *(Practice only)* | Mindful breathing A | Chronic pain |
| **3** | Audio | *(Practice only)* | Mindful breathing A | Light exposition and health |
| **4** | Video | Arriving in presence | Mindful breathing B | Sleep disturbances |
| **5** | Audio | *(Practice only)* | Mindful breathing B | Body memory |
| **6** | Audio | *(Practice only)* | Mindful breathing B | Migraine |
| **7** | Video | Arriving in the body | Bodyscan A | Burnout |
| **8** | Audio | *(Practice only)* | Walking meditation | Equanmity* |
| **9** | Audio | *(Practice only)* | Bodyscan A | Social inequality and health |
| **10** | Video | Subjectivity of perception | Bodyscan B | Sore muscles / vegan diet |
| **11** | Audio | *(Practice only)* | Walking meditation | Happiness* |
| **12** | Audio | *(Practice only)* | Bodyscan B | Time perception |
| **13** | Video | Communicating mindfully | Mindful attention to body sensations | Gender-specific health |
| **14** | Audio | *(Practice only)* | Mindful attention to body sensations | Illness as language of the soul* |
| **15** | Audio | *(Practice only)* | Mindful attention to body sensations | Aging |
| **16** | Video | Non-judgement | Mindful attention to body sensations | Sugar |
| **17** | Audio | *(Practice only)* | Mindful listening | Medicinal plants* |
| **18** | Audio | *(Practice only)* | Mindful listening | Self-deceit |
| **19** | Video | Dealing with stress | Mindfully approaching emotions | Maintaining health* |
| **20** | Audio | *(Practice only)* | Mindfully approaching emotions | Migration and health |
| **21** | Audio | *(Practice only)* | Mindfully approaching emotions | Epigenetics |
| **22** | Video | Turning towards instead of turning away | Turning towards instead of turning away | Sensible footwear |
| **23** | Audio | *(Practice only)* | Approaching unpleasant feelings | Obsessive-compulsive disorder |
| **24** | Audio | *(Practice only)* | Awareness of thinking | Self-efficacy |
| **25** | Video | Positive qualities | Loving kindness | Microorganisms* |
| **26** | Audio | *(Practice only)* | Loving kindness | Cardiovascular diseases |
| **27** | Audio | *(Practice only)* | Loving kindness | Hypnotherapy |
| **28** | Video | Decentring | Open monitoring | Staying active in the office |
| **29** | Audio | *(Practice only)* | Open monitoring | Negative empathy |
| **30** | Audio | *(Practice only)* | Silent meditation | Pain perception |
| **31** | Video | Reflecting the course | Silent meditation | Physical activity |

**Table S1:** *Contents and delivery scheme of MMT and HT. MMT included both theoretical and practical instructions, whereas HT was limited to delivering informative content. Contents that were replaced from prior versions of the HT are marked with an asterisk. (MMT = Mindfulness Meditation Training; HT = Health Training)*

**Figure S2:** *Clusters resulting from the seed-based analysis using hypothalamic (A) and insular (B) seed regions. Regions with increased functional connectivity are depicted in red, decreases in functional connectivity are depicted in blue.*

| **Scale** | **HT** | | **MMT** | |  |  |  |
| --- | --- | --- | --- | --- | --- | --- | --- |
|  | *M* | *SD* | *M* | *SD* | *t* | *df* | *two-sided p* |
| SEES | 61.8 | 1.8 | 64.3 | 1.2 | - 1.130 | 56 | 0.263 |
| SSES | 32.4 | 1.7 | 33.7 | 1.4 | - 0.613 | 64 | 0.542 |
| FCQT | 79.4 | 6.1 | 84.0 | 5.2 | - 0.575 | 64 | 0.567 |
| MAAS | 55.5 | 2.2 | 53.9 | 1.8 | 0.555 | 64 | 0.581 |
| RS | 17.0 | 1.1 | 14.7 | 0.9 | 1.732 | 64 | 0.088 |

**Table S3:** *Results of t-tests for independent samples conducted on behavioral measures obtained at baseline. (SEES = Salzburg Emotional Eating Scale, SSES = Salzburg Stress Eating Scale, FCQT = Food Cravings Questionnaire – Trait, MAAS = Mindful Attention and Awareness Scale, RS = Restraint Scale)*

| **Hypothalamus** | **Cluster region** | **MAAS** | | **FCQT** | | **SEES** | | **SSES** | |  |
| --- | --- | --- | --- | --- | --- | --- | --- | --- | --- | --- |
| L. Lateral | L. PreSMA | *βₛ* | 0.23 | *βₛ* | 0.04 | *βₛ* | - 0.05 | *βₛ* | 0.02 | |
|  |  | *p* | 0.23 | *p* | 0.88 | *p* | 0.86 | *p* | 0.94 | |
| R. Lateral | L. Ventral PCC | *βₛ* | 0.15 | *βₛ* | 0.28 | *βₛ* | - 0.24 | ***βₛ*** | **- 0.43** | |
|  |  | *p* | 0.74 | *p* | 0.23 | *p* | 0.32 | ***p*** | **0.03** | |
| L. Medial | L. Striatum/Thalamus | *βₛ* | - 0.02 | ***βₛ*** | **0.61** | *βₛ* | - 0.27 | *βₛ* | - 0.08 | |
|  |  | *p* | 0.93 | ***p*** | **< 0.01** | *p* | 0.27 | *p* | 0.69 | |
| L. Medial | R. Precuneus | ***βₛ*** | **0.33** | *βₛ* | - 0.28 | *βₛ* | 0.28 | *βₛ* | 0.04 | |
|  |  | ***p*** | **0.05** | *p* | 0.21 | *p* | 0.24 | *p* | 0.82 | |
| L. Medial | R. Caudal Precuneus | *βₛ* | - 0.13 | *βₛ* | 0.21 | *βₛ* | 0.17 | *βₛ* | - 0.01 | |
|  |  | *p* | 0.48 | *p* | 0.93 | *p* | 0.52 | *p* | 0.98 | |
| L. Medial | R. Angular Gyrus | ***βₛ*** | **- 0.38** | ***βₛ*** | **- 0.46** | *βₛ* | 0.12 | *βₛ* | 0.03 | |
|  |  | ***p*** | **0.03** | ***p*** | **0.05** | *p* | 0.62 | *p* | 0.88 | |

**Table S4:** *Results of linear regression between the change in functional connectivity and the change in behavioral measures. (MAAS = Mindful Attention and Awareness Scale, FCQT = Food Cravings Questionnaire – Trait, SEES = Salzburg Emotional Eating Scale, SSES = Salzburg Stress Eating Scale, R = Right hemispheric; L = Left hemispheric, PreSMA = Pre-supplementary motor area, PCC = Posterior cingulate cortex)*
